# Flow Cytometry in the Diagnosis of Canine T-Cell Lymphoma

**DOI:** 10.3389/fvets.2021.600963

**Published:** 2021-04-21

**Authors:** Stefano Comazzi, Fulvio Riondato

**Affiliations:** ^1^Dipartimento di Medicina Veterinaria, Università degli Studi di Milano, Lodi, Italy; ^2^Dipartimento di Scienze Veterinarie, Università degli Studi di Torino, Grugliasco, Italy

**Keywords:** dog, lymphoma, T-cell, flow cytometry, diagnosis, prognosis

## Abstract

T cell lymphoma (TCL) is a heterogenous group of lymphoid malignancies representing about 30–40% of all canine lymphomas and often harboring a very aggressive behavior. WHO classification identifies the majority of TCLs as peripheral TCL, but other subtypes with peculiar presentation and outcome have been recognized. This review aims to explore the use of flow cytometry for refining the diagnosis of canine TCL, putting a particular emphasis on the identification of some peculiar immunotypes, such as T zone lymphoma; on the investigation of putative prognostic markers; and on the evaluation of lymphoma stage and of the minimal residual disease.

## Introduction

T cell lymphoma (TCL) is a heterogeneous group of lymphoid neoplasms stemming from T cells. It represents about 30–40% of canine lymphomas. Prevalence of B vs. TCLs varies among different breeds with TCL being highly prevalent in Irish Wolfhound, Shih Tzu, Airedale Terrier, Cavalier King Charles Spaniel, Yorkshire Terrier, Siberian Husky, and some other breeds (1).

Solid presentation with enlarged lymph nodes or the increased volume of any other lymphoid organ are distinctive traits; these features easily allow a distinction between lymphoma and leukemia, such as T chronic lymphocytic leukemia, which, on the contrary, originates from bone marrow or spleen. Despite highly variable cytological aspects, most TCLs fall under the umbrella of peripheral T cell lymphoma (PTCL), not otherwise specified (NOS), according to WHO classification (2). T-zone lymphoma (TZL) and T lymphoblastic lymphoma (LL) are two other commonly found TCL morphotypes, whereas enteropathy-associated TCL, cutaneous epitheliotropic TCL, mediastinal lymphoma, and hepatocytotropic/hepatosplenic lymphoma constitute uncommon findings. The updated Kiel classification identifies several different cytological subtypes of TCL, thus confirming their extremely heterogeneous morphology (3) (Table 1). According to this scheme, TCL could be further divided into low-grade TCLs, characterized by indolent behavior, and high-grade TCLs, characterized by a very aggressive course with median survival time statistically lower than its B cell counterpart (4).

**Table 1:** Possible correlation between updated Kiel classification and WHO classification for TCLs according to Ponce et al. (3).

**Updated Kiel classification**		**WHO classification**
**PRECURSOR T CELL LYMPHOMA**		
	Lymphoblastic lymphoma	Precursor T cell lymphoblastic lymphoma/leukemia
**MATURE T CELL LYMPHOMA**		
Low-grade	Prolymphocytic	T cell chronic lymphocytic leukemia/prolymphocytic leukemia
	Pleomorphic small cells	Peripheral T cell Lymphoma, unspecified
	Small clear cell/T zone lymphoma	Peripheral T cell Lymphoma, unspecified
High-grade	Pleomorphic mixed	Peripheral T cell Lymphoma, unspecified
	Pleomorphic large cell	Peripheral T cell Lymphoma, unspecified
	Immunoblastic	Peripheral T cell Lymphoma, unspecified
	Plasmocytoid	Peripheral T cell Lymphoma, unspecified
	Aggressive large granular	Enteropathy-type T cell lymphoma OR natural killer leukemia OR extranodal nasal-type
**CUTANEOUS LYMPHOMA**		
	Cutaneous T cell, low-grade	Mycosis fungoides/Sezary syndrome
	Cutaneous T cell, high-grade	Cutaneous T cell lymphoma

## Identification of T Cell Lineage

Several antibodies have been generated to recognize specific canine lymphoid markers and to define normal T cell subpopulations *via* flow cytometry (FC). Antibodies against surface epitopes are highly preferable for FC. Antibodies capable of recognizing intracellular epitopes represent another option, but they do require one or more additional permeabilization steps that may alter cell morphology. This said, in the absence of any definitive reactivity to membrane antigens, intracellular labeling for cytoplasmic CD3 can be performed to confirm a T cell origin. Antibodies prelabeled with different fluorochromes are recommended to identify co-expressions and to reduce the number of cells needed for a complete antibody panel. Common antibodies used to phenotype T cell markers and expected reactivity in non-neoplastic cells are reported in Table 2.

**Table 2:** Common antibodies used for the characterization of T cells in dog and expected reactivities.

**Target**	**Antibody clones**	**Reactivity**
CD3	CA17.2A12	T cells
CD3cy	CD3-12	T cells
CD5	YKIX322.3	T cells and a subset of B cells
CD4	YKIX302.9	T-helper cells
	CA13.1E4	
CD8α	YCATE55.9	T-cytotoxic cells
	CA9.JG3	
CD8β	CA15.4G2	T-cytotoxic cells
CD45	YKIX716.13	All leukocytes
	CA12.10C12	
CD44	IM7	All hematopoietic cells
CD18	CA1.4E9	All leukocytes
MHC II	YKIX334.2	Monocytes, Histiocytes, Lymphocytes
	CA2.1C12	
TCR αβ	CA15.8G7	Most T cells
TCRγδ	CA20.6A3	Gamma-delta T cell subset
	CA20.8H1	
CD25	P4A10	Activated lymphocytes
	ACT-1	
CD11d	CA11.8H2	Splenic T cells and histiocytes
FoxP3	FJK-16s	Activated cells, T-Reg cells
Ki67	MIB-1	Proliferating cells

T-helper cells are the most frequent lymphocyte subset in canine lymph nodes (5) and can be easily recognized for their positivity to CD4, CD3, CD5, and MHC II and for their prevalent positivity to TCRαβ and, if activated, to CD25. A peculiar subset of CD4+ T-regulatory cells (T-regs), representing <1% of lymphocytes in circulating blood and <5% in lymph nodes, can be identified for their high expression of CD25 and for the co-expression of nuclear antigen Fox-P3 (6).

T-cytotoxic cells are less frequent in lymph nodes, spleen, and peripheral blood and may be easily identified for positivity to CD8, CD3, CD5, and MHC II.

In the thymus, besides mature T-helper and T-cytotoxic cells, a mixed population of immature T cells co-expressing CD4 and CD8 (double-positive thymocytes) or lacking both antigen expression (double-negative T cells) are found.

Less frequently, T cells may express CD11d and/or TCRγδ. These phenotypes are often suggestive of splenic or, less frequently, intestinal origin (7). These cells, in some instances, can feature a large granular lymphocyte (LGL) morphology (8).

## Differentiating Reactive and Neoplastic T Cells

When it comes to differentiating between reactive and neoplastic T cell populations, distinguishing paracortical hyperplasia from TZL represents the most challenging step (9). Paracortical hyperplasia is an infrequent transitory hyperplastic condition due to chronic dermatitis or to some specific antigens stimulating T response (such as in Leishmaniosis or in viral infections). It generally represents the immune response first phase, and it precedes plasma cell proliferation (10). Differentiation *via* cytology may be inconclusive, and FC should be considered the most rapid and effective way to differentiate. This is due to the very peculiar phenotype of TZL (CD45-CD5+CD21+) that can be easily detected using a multicolor approach (11, 12).

Differentiating between high-grade TCL and reactive lymph node hyperplasia tends to be easier because hyperplastic lymph nodes (with follicular and/or lympho-plasmacellular hyperplasia) are generally composed of a mixed population of T and B cells, sometimes with a slight increase of large B cells. The T cell population is generally composed of a mixed population of both CD4 positive and CD8 positive cells, suggesting polyclonal expansion. On the contrary, most PTCL feature a homogeneous expansion of a single T cell subpopulation. The presence of medium to large T lymphoid cells with forward scatter (FSC) higher than 1.3–1.5 times that of normal lymphocytes also suggests lymphoma. Residual non-neoplastic B and T cells are generally few, and this supports the hypothesis of a clonal expansion, confirming lymphoma.

Neoplasia also may be confirmed through the identification of phenotypic aberrancies. Qualitative and quantitative aberrancies are frequent, occurring in more than 75% of TCLs (13). The most frequent phenotypic aberrancies are loss of CD5 or CD3 expression; loss or co-expression of both CD4 and CD8; marked decrease/loss of MHC class II expression; loss of CD45 or other pan-leukocyte antigen expression; and concurrent expression of a B-specific antigen, such as CD21 or CD79. The use of wide antibody panels increases the odds of detecting aberrant phenotypes, which may be considered a hallmark of neoplasia (13).

When mediastinal masses are composed of a lymphocyte-rich population, FC of the lymphoid population is considered an excellent tool to distinguish mediastinal lymphoma from non-neoplastic normal lymphocytes, thus supporting thymoma. A cutoff value of 10% of double positive CD4+CD8+ thymocytes and small cell size (similar to that of a circulating lymphocyte) is strongly supportive of thymoma (14).

## Defining Immunophenotype of Canine Lymphoma

Immunophenotype is a widely accepted prognostic feature in canine high-grade lymphoma (4, 15, 16). Immunohistochemistry is the traditional tool performed to define immunophenotype, but FC has recently gained popularity due to the availability of wide antibody panels, its fast response and the possibility of detecting co-expressions and of accurately quantifying antigen expressions (17).

Consistent approaches and cutoff values to define TCLs, however, are still lacking. Wilkerson et al. (18) considered TCLs if 60% of the population expressed any T cell antigen. A more recent study considered as inclusion criteria to define TCL a lymph node with a cytology/histopathology suggestive of lymphoma together with a positive reactivity to CD3, or CD4, CD8, in more than 10% of large lymphoid cells (19). In another study, more than 65% of large lymphoid cells or more than 80% of total lymphoid cells being positive to CD4 were considered essential to define CD4+ TCLs (20).

In a recent study on CD8+ and CD4–CD8– lymphomas, TCL cases were identified by a discrete majority population of T cells with a homogeneous phenotype or a population of T cells showing at least one feature of aberrancy (21). In another study from the same group, the T cell phenotype was defined upon the expression of CD3 or CD5 with variable expression of T cell subset antigens CD4 and CD8 (22); no cutoff values were reported.

Considering the abovementioned papers, despite the lack of a consensus, TCL is likely to be correctly diagnosed if a lymph node presents with cytology/histology suggestive of lymphoma and if (1) a prevalent population of medium-to-large lymphoid cells (≥1.3 times the size of normal T-lymphocytes) expresses uniformly CD3 and/or CD5; (2) more than 80% of total lymphoid cells have a homogeneous expression of CD4 or CD8; or (3) any aberrant phenotype in CD3 or CD5 positive cells is present, including the loss of membrane expression of CD3 in cells retaining cytoplasmic expression of CD3 only.

In rare instances, co-expression of T and B lineage-specific markers may occur. More specifically, CD21 may be expressed in TZL, and CD79 may be aberrantly expressed in some PTCLs. A comprehensive evaluation of a complete panel of anti-T antibodies is normally enough to differentiate B cells from TCLs with an aberrant expression of B cell markers, but in some cases, some other ancillary tests (PARR and histopathology) might be necessary although caution should be made using PARR as an immunotyping method (23).

## Refining the Diagnosis of TCL Subtypes

Flow Cytometry is a valid and rapid tool not only to define T cell lineage, but also to refine the classification of TCL subtypes in addition to cytomorphology and histopathology.

### Peripheral T Cell Lymphoma

**Figure 1:**
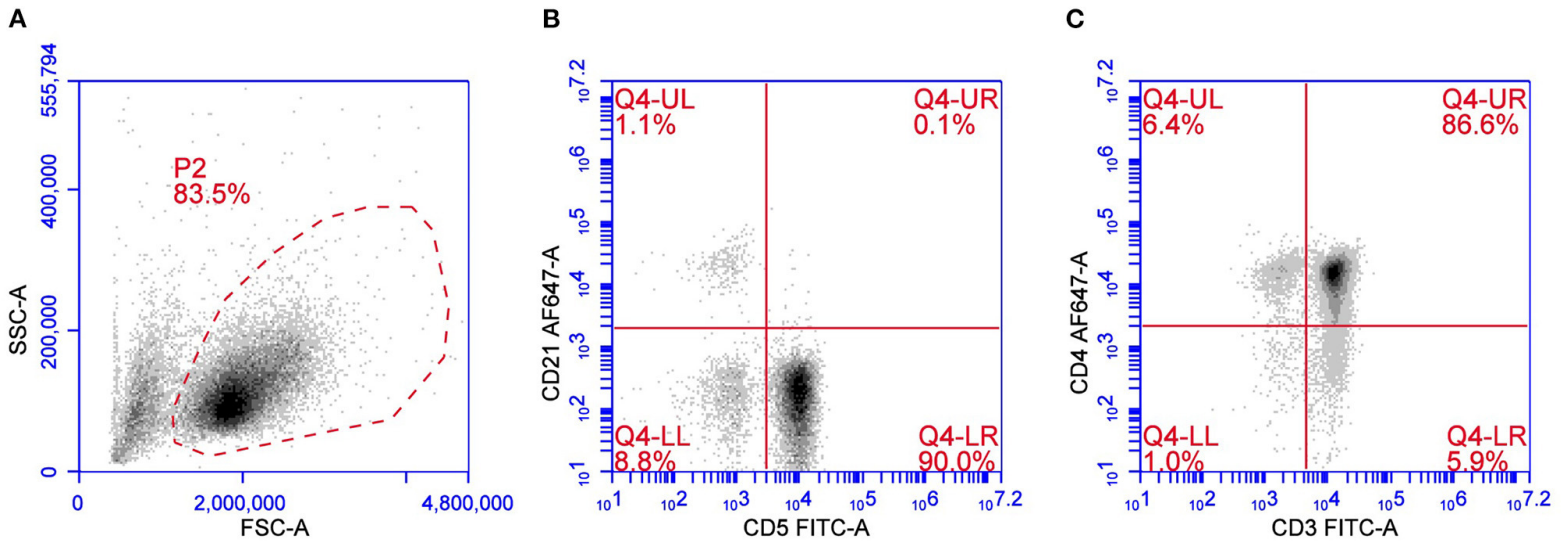
Flow cytometric presentation of a PTCL. **(A)** Forward scatter (FSC) vs. side scatter (SSC) plot after doublet exclusion showing a unique population of medium-to-large sized cells. **(B,C)** CD5 vs. CD21 plot **(B)** and CD3 vs. CD4 plot **(C)** of P2-gated cells showing a predominant population of CD5+CD3+CD4+ cells.

Peripheral T cell lymphoma is the most widely distributed TCL subtype and comprises a heterogeneous group of different immunotypes with different cytological presentations and variable outcomes. According to the updated Kiel classification scheme adapted to the dog, PTCLs are characterized by six morphological subtypes, namely pleomorphic small-cell, small clear-cell, pleomorphic mixed, pleomorphic large-cell, immunoblastic, and plasmacytoid lymphomas (3). Among PTCLs, the most frequent phenotype is CD45+CD3+ CD4+MHC II–/low (Figure 1) (19, 20). In all PTCL subtypes, CD5 expression is usually positive, but a loss of CD5 and CD3 may aberrantly occur and have a possible prognostic meaning. CD25 is generally negative, suggesting that they are inactive cells. TCRαβ is highly prevalent. This immunophenotype occurs in 43–45% of multicentric TCLs according to two different studies (19, 22). Forward scatter properties show a medium-to-large size with median FSC 1.4 times greater than in non-neoplastic lymphocytes (22). This subtype exhibits a consistent gene expression profile with the positive regulation of phosphatidylinositol 3-kinase (PI3K) activity. Phosphatidylinositol 3-kinase works together with AKT and mTOR to regulate the cell cycle, leading to increased cell proliferation and survival. The AKT/PI3K/mTOR signaling axis is usually antagonized by the product of phosphatase and tensin homolog gene (PTEN), a tumor suppressor gene that is downregulated in CD4+ TCL as well (22).

The less frequent immunophenotypes of PTCL are CD45+CD3+CD8+MHC II– and CD45+CD3+CD8–CD4–MHCII– comprising about 11.9 and 13.5% of TCLs, respectively (19).

A recent study based on 119 cases demonstrated that the former immunophenotype is often associated with a cutaneous localization, whereas the latter more frequently affects mediastinal lymph nodes (21). Forward scatter properties are similar between the two subtypes, with median values of FSC >1.2 times in neoplastic cells than in normal lymphocytes.

Other possible immunotypes are rare, but may include CD3+CD4+CD8+, CD3+CD4+MHC II+, and CD3+CD8+MHCII+ (19). However, specific studies on these immunotypes including histopathology and the evaluation of CD45 expression to exclude TZL are still lacking.

### Lymphoblastic Lymphoma

Lymphoblastic lymphoma is a rare subtype identified in about 10% of TCL cases and about 3% of all canine lymphoma (3). According to the WHO classification, LL is considered a solid variant of T cell acute LL and is generally classified as a neoplasm arising from precursor cells. Despite the homogeneous population of small cells without an evident nucleolus, LL is an aggressive high-grade lymphoma, and the mitotic index is often very high. The outcome is generally poor without any statistical difference compared with CD4+ PTCL. One study (24) described a high prevalence of LL in the boxer breed, harboring a less aggressive course and a longer survival. A recent study on CD4+ TCL reported an overlapping of gene expression profiles between PTCL and LL in dogs (22). In terms of FC, the prevalent immunophenotype of LL is similar to that of PTCL (CD45+CD3+CD4+MHC II–), and despite the supposed origin from precursor cells, CD34 is generally negative. Terminal deoxynucleotidyl transferase (TdT), which is often used to confirm lymphoblastic nature in human LL (25), has not yet been optimized for dogs.

In cases characterized by small-sized cells and no expression of precursor markers, the FC determination of proliferative activity can be useful to grade the tumor. A significant difference between low- and high-grade lymphomas, according to the updated Kiel classification, has been shown both for Ki67 expression (26) and S-phase fraction (27), and a cutoff of 12.2 and 3.15% was proposed, respectively, independent of the lineage. Nevertheless, in the study by Poggi et al. (26), T cell high-grade lymphomas presented higher Ki67 values compared with B cell high-grade forms (45.0 vs. 29.3%).

### T-Zone Lymphoma

**Figure 2:**
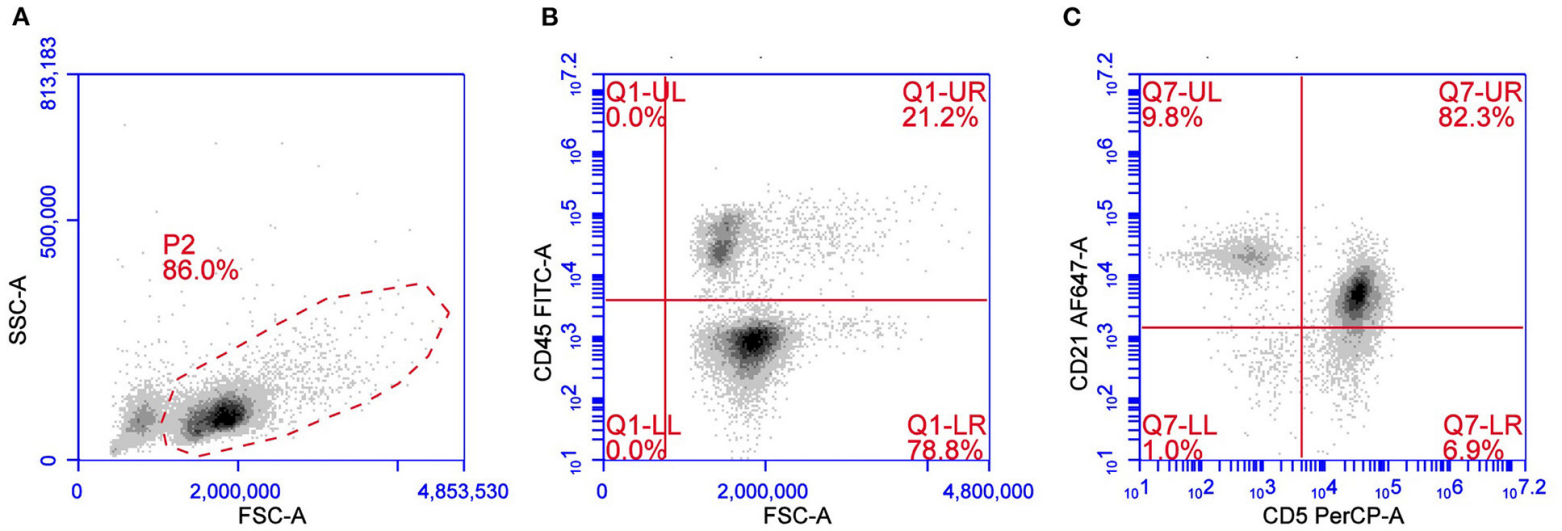
Flow cytometric presentation of a TZL. **(A)** Forward scatter (FSC) vs. side scatter (SSC) plot after doublet exclusion showing a prevalent population of medium sized cells. **(B,C)** FSC vs. CD45 plot **(B)** and CD5 vs. CD21 plot **(C)** of P2-gated cells showing that medium-sized cells are CD45–CD5+CD21+dim.

T-zone lymphoma is a low-grade indolent lymphoma occurring in about 10% of TCL. Cytologically TZL can be described as a lymphocytic, small clear-cell lymphoma. Neoplastic cells are easily recognized using a multicolor approach for their characteristic loss of CD45 and for the frequent aberrant expression of CD21, a B cell antigen, in the context of a CD3+CD5+MHC II+ lymphoma (11, 12) (Figure 2). Although rare, loss of CD45 expression may also occur in other lymphoma subtypes besides TZL (28). CD25 is frequently expressed in TZL, thus confirming the neoplastic cell activation status. Expression of CD4 and CD8 may be variable (CD4+CD8–, CD4–CD8+, CD4–CD8–, or CD4+CD8+) without any evident correlation with outcome. Due to the very peculiar phenotype, TZL also may be easy to recognize in its earlier phases, when a mixed population of residual non-neoplastic lymphocytes is still present, in cases of concurrent lymphomas in the same node (29–31) or, again, when they are detected in non-lymphoid tissues, such as peripheral blood and bone marrow, which are infiltrated in more than 90% of cases (32). The low proliferative activity reported by TZLs is described in two studies focusing on B and TCLs and supports the low aggressiveness of the tumor (26, 27). Several studies confirm the indolent behavior of this subtype, making aggressive chemotherapy unnecessary in most dogs with TZL (4, 11, 32, 33).

Studies on FC aspects in dogs with other TCL subtypes are fragmentary and based on very limited caseloads. Limited data are due to the frequent extranodal localizations that impair the overall quality of FC samples. According to a study, in fact, <60% of extranodal lymphoma were suitable for FC processing, compared with more than 90% of nodal ones (34). Immunohistochemistry and other techniques are more suitable to investigate this kind of lesions.

### Hepatosplenic/Hepatocytotropic Lymphomas

Keller et al. (35) described a series of nine cases of hepatosplenic/hepatocytotropic lymphomas. Immunophenotype was assessed *via* FC, and immunohistochemistry and neoplastic cells expressed CD3+ (5/7), TCRαβ- (5/5), TCRγδ+ (3/5), CD11d+ (6/7). CD8α was inconsistently expressed in 4/6 cases, and CD4 and CD8β were not expressed in any of the cases. The positivity to CD11d suggests an origin from the splenic red pulp and may help to differentiate hepatosplenic from hepatocytotropic lymphomas; these findings are consistent with what has been described in human medicine (36).

### Cutaneous Epitheliotropic T Cell Lymphoma

Cutaneous Epitheliotropic T Cell Lymphoma (CETL) is commonly investigated *via* immunohistochemistry because cutaneous lesions are difficult to sample for FC and the presence of non-neoplastic reactive lymphocytes may affect FC interpretation. Flow cytometry may be useful to evaluate immunophenotype in infiltrated lymph nodes from primary CETL or in peripheral blood if Sezary syndrome is present (37). In these cases, neoplastic cells are CD3+CD8+TCRγδ+ and frequently lack CD5 expression (38).

### Mediastinal Lymphoma

Flow cytometric features from seven cases of mediastinal lymphoma were reported in a small case series (14). Four out of seven cases expressed CD4; one expressed only CD45, CD34, and CD14 and exhibited T cell clonality by PARR analysis, and one was of B cell origin. The last case was double positive for CD4+CD8+ but was mainly composed of large cells. The authors concluded that FC is a useful tool for discriminating mediastinal masses and that thymoma could be ruled out in all cases in which <10% of the small lymphocytes were CD4+CD8+. However, a more recent paper (39) describes expansion of CD4–CD8– cells in a case of canine thymoma, partially contradicting the previously published paper.

### Enteropathy-Associated T Cell Lymphoma

Enteropathy-associated T Cell Lymphoma (EATL) is generally investigated *via* immunohistochemistry and shows positivity to CD3 and occasionally to CD30, a marker of anaplastic lymphoma in humans (40). In rare instances, aberrant co-expression of B cell antigen CD20 is possible (41). To the best of our knowledge, no FC studies in EATL are available in dogs.

## Refining the Prognosis of T Cell Lymphoma

Outcomes of TCLs vary from poorly aggressive TZL with an indolent course (the majority of cases) to highly aggressive PTCL and hepatosplenic lymphoma. Being that TCLs comprise a wide spectrum of different entities, each of them featuring a different biological behavior and outcome, an accurate definition of the immunophenotype is crucial for predicting outcome and setting up a tailored therapy. Even though specific studies on prognosis in TCLs are fragmentary, different subtypes could likely have different prognostic factors.

Flow Cytometry is likely to be the best available tool to accurately detect TZL given the very peculiar immunophenotype and the loss of CD45 expression. The presence of CD45–CD5+ cells in lymph nodes and, in most cases, in peripheral blood allows the suspicion of TZL also in its earlier stages, despite the lack of a marked lymphoadenomegaly in palpable lymph nodes. To date, no specific immunophenotypic prognostic markers for TZL have been described, but median survival time is reported to range between 637 and 930 days according to different studies (11, 12, 42). However, some aggressive cases featuring a similar immunophenotype but leading to short survival time are sometimes described (28, 43).

Peripheral T cell lymphomas are generally associated with short survival times [162 days according to (15)]. A recent study defined shorter survival times and progression-free intervals (PFI) if CD4+MHC II– phenotype is found (160 and 108 days, respectively) (19). Unfortunately, CD45 expression was not included in this study, and the inclusion of some TZLs (expressing high MHC II) in the caseload may have contributed to bias in the comparison. In another study (22), no differences were found in survival times among CD4+MHCII–, CD8+MHCII–, and CD4–CD8–MHCII–, but large cell size was associated with a significantly shorter PFI and overall survival (20, 22). CD5 expression (20) or loss of CD3 expression or co-expression of CD79a (44) were also moderately associated with shorter survival time in CD4+MHC II– PTCLs.

Considering all these findings, FC immunophenotyping seems to be useful to better refine the subtype of TCL and to differentiate PTCL from TZL. Furthermore, large size and aberrant loss of CD3, or aberrant expression of CD79 may be linked to a more aggressive course.

In addition to defining the degree of malignancy, Ki67 values have proven useful for stratifying high-grade B cell lymphomas with different prognosis (45). Different levels of Ki67 expression were also described within high-grade TCLs (26). However, whether it can represent a prognostic index for TCLs has yet to be determined.

## Staging T Cell Lymphoma

Flow cytometry is an effective tool for defining infiltration of neoplastic cells in different tissues, mainly in liquid ones such as peripheral blood (PB) and bone marrow (BM). The impact of the stage on the prognosis of canine lymphoma is not consistent among different studies, and the current findings are often influenced by the inclusion of different lymphoma subtypes. Also, the strategy to detect infiltration (microscopic evaluation, immunohistochemistry, FC, PARR) and the cutoff values used vary among studies, thus strongly affecting results. Some studies, however, suggest that stages, in particular, stage V (i.e., infiltration of PB and/or BM or any other extra lymphoid tissue) may affect outcomes differently in different subtypes. Most studies on this issue have been focused on B cell lymphomas (46, 47), and no specific studies on the prognostic significance of PB and BM infiltration are available when it comes to TCLs.

As described, PTCLs are a very heterogeneous group in terms of immunophenotype and morphology, suggesting that the strategies used to detect neoplastic infiltration in peripheral and medullary blood may differ. Multicolor FC may help to easily detect the percentage of CD3+CD4+MHC II– neoplastic cells in PB and BM and to distinguish this circulating neoplastic cells from residual non-neoplastic T-helper cells (MHC II+). Similarly, CD3+CD8+MHC II–, CD3+CD4–CD8–, or CD3+CD4+CD8+ lymphomas may be accurately quantified using a multicolor approach. Other strategies may be used if aberrant patterns are expressed, such as loss of CD3 or CD5 expression. In these cases, double labeling for CD3 and CD5 may easily identify neoplastic cells. In contrast, differentiation between neoplastic and residual circulating lymphocytes can be challenging if the immunophenotype of neoplastic cells is similar to that of normal T-helper (CD4+MHC II+) or T-cytotoxic (CD8+MHC II+) lymphocytes and no aberrancies are present. In these instances, a larger cell size compared with that of circulating lymphocytes can help to suspect neoplastic infiltration.

To the best of our knowledge, no specific studies (featuring a consistent caseload of PTCLs) on the prognostic significance of PB or BM infiltrations are available, but some findings suggest that the presence of leukemic cells is related to a bad outcome in TCL (19), thus suggesting a possible prognostic role. This said, cutoff values to define leukemic infiltration were not specified.

T-zone lymphoma is associated with the presence of neoplastic circulating cells in more than 90% of cases (32) with or without lymphocytosis. BM is often mildly or not infiltrated, and cytopenia is generally scarce. Neoplastic circulating cells are easy to detect using multicolor FC and double labeling for CD45/CD5. Sometimes their detection in PB may precede the development of an evident solid mass. Despite the frequent peripheral infiltration, the prognosis of TZL is generally quite good, and no data support a prognostic role of PB or BM infiltration to date.

## Evaluation of Minimal Residual Disease

From a technical standpoint, the same strategies used to stage TCLs can be used to evaluate the effects of chemotherapy and to monitor the minimal residual disease (MRD). Minimal residual disease *via* FC has been suggested as a possible feasible method to monitor the effect of chemotherapy and to predict relapse in canine diffuse large B cell lymphoma (48). At present, however, no specific studies on the evaluation of MRD *via* FC and its possible prognostic role in canine TCL are available.

## Future Perspectives

Although the availability of specific antibodies for most T cell subpopulations improved the diagnosis of TCL, there is still a lack of specific antibodies for some peculiar subtypes, such as natural killer cells, precursor T cells, and/or antibodies that may potentially be useful to better understand the prognosis and peculiar risk factors. Again, the commercial availability of antibodies labeled with more different fluorochromes would improve the multicolor approach, allowing detection also of a rare population of cells.

Also, the clinical significance of staging PB and BM infiltration of TCLs is far from being completely understood, and multi-institutional prospective studies with a standardized approach and therapy are needed to obtain adequate statistic power.

## Author Contributions

All authors have made a substantial, direct and intellectual contribution to the work, and approved it for publication.

## Conflict of Interest

The authors declare that the research was conducted in the absence of any commercial or financial relationships that could be construed as a potential conflict of interest.
